# Observational Study: Lung Function and Symptom Control in Youth With Asthma up to 34 Months After COVID‐19

**DOI:** 10.1002/ppul.71288

**Published:** 2025-09-09

**Authors:** Kristina Gaietto, Nicholas Bergum, Daniel J. Weiner, Erick Forno

**Affiliations:** ^1^ Department of Pediatrics, Division of Pulmonology University of Pittsburgh School of Medicine Pittsburgh Pennsylvania USA; ^2^ University of Pittsburgh School of Medicine Pittsburgh Pennsylvania USA; ^3^ Department of Pediatrics, Division of Pulmonology Indiana University of School of Medicine Indianapolis Indiana USA

**Keywords:** child, pediatrics, SARS‐CoV‐2

## Abstract

**Introduction:**

Prior studies of pediatric asthma control and lung function after COVID‐19 have been limited by short follow‐up intervals. We aimed to evaluate symptom control and lung function in children with asthma up to 34 months post‐COVID‐19.

**Methods:**

We conducted a prospective observational chart review study. We reviewed electronic health records of children with asthma in the Western Pennsylvania COVID‐19 Registry, abstracting pre‐ and all post‐infection spirometry results and Childhood Asthma Control Test (C‐ACT) or Asthma Control Test (ACT) scores (to measure symptom control) through August 2023. We conducted adjusted mixed models with linear spline to compare C‐ACT/ACT or FEV_1_ before and after COVID‐19. For individuals with worse outcomes at initial follow‐up, we evaluated characteristics associated with lack of eventual recovery.

**Results:**

We found no significant differences between baseline and post‐infection symptom control (*n* = 267) or lung function (*n* = 196). Of the 28% of children who had worse lung function at initial follow‐up, 34% fully recovered at final follow‐up. Of the 19% with worse C‐ACT/ACT score at initial follow‐up, 38% fully recovered at final follow‐up. Final follow‐up median C‐ACT/ACT scores and mean FEV_1_ were in the normal range even for the group without eventual recovery. Obesity (*p* = 0.04) was associated with hindered symptom control recovery.

**Conclusion:**

There were no significant differences between baseline and follow‐up symptom control or lung function in children with asthma up to 34 months post‐COVID‐19. Only a small proportion of children worsened and did not recover, and decrements were generally small. Obesity was associated with impaired symptom control recovery.

## 1 Introduction

The coronavirus disease (COVID‐19) pandemic began in March 2020. Though there was initially great concern that individuals with asthma would be at greater risk of infection, severe disease, and death than healthy peers, most early studies of COVID‐19 and asthma ultimately did not support this initial hypothesis [1, 2, 3, 4, 5, 6]. However, longer‐term effects of SARS‐CoV‐2 infection remain largely unknown, given that the pandemic onset was still relatively recent at the time of those analyses. It is only now possible to begin studying longer‐term outcomes for youth and adults with asthma following SARS‐CoV‐2 infection to assess for later negative effects of prior infection.

We previously conducted a registry‐based prospective study of children aged 6−21 years with asthma, comparing pre‐ and post‐SARS‐CoV‐2 infection lung function (*n* = 114) and asthma symptom control (*n* = 171). We found that overall, a median of ~5 months after SARS‐CoV‐2 infection, there were no significant differences between pre‐infection baseline and post‐infection follow‐up lung function or asthma symptom control [7]. However, a subgroup of individuals did have worse lung function (29.7%) or asthma symptom control (17.5%) at follow‐up. Compared to children whose asthma control did not worsen after COVID‐19, those whose asthma control worsened had shorter time to follow‐up and were more likely to have had an asthma exacerbation during acute infection. We acknowledged that longer‐term studies would be needed to test for later effects of COVID‐19 in youth with asthma and to follow the subgroup of children with worse post‐infection symptom control or lung function.

Our present study expands upon our previous findings. We re‐reviewed the electronic health records (EHR) for youth in our COVID‐19 registry who had pre‐existing asthma and pre‐infection (baseline) asthma symptom control or lung function data, to assess longer‐term post‐COVID‐19 asthma symptom control and lung function. We examined outcomes up to 34 months after acute SARS‐CoV‐2 infection. We also conducted subgroup analyses among children with worse asthma symptom control or lung function at initial follow‐up to assess for characteristics associated with subsequent recovery (or lack thereof).

## 2 Methods

### 2.1 Study Population and Data Collection

Cases were identified from the Western Pennsylvania COVID‐19 Registry, which has been previously described [8, 9]. In brief, the Western Pennsylvania COVID‐19 Registry was established in spring 2020 to record baseline characteristics, acute infection details, and outcomes of children infected with SARS‐CoV‐2 who presented to the University of Pittsburgh Medical Center (UPMC) Children's Hospital of Pittsburgh (CHP) and its affiliated primary care network. Children ages 0−21 years old with a positive SARS‐CoV‐2 polymerase chain reaction or antigen test result were included in the registry. A multidisciplinary team of pediatricians, pediatric specialists, advanced practice providers, and nurses identified pediatric COVID‐19 cases. The team manually abstracted patient demographics (e.g., age, sex, race, body mass index [BMI], baseline comorbidities) and details of acute COVID‐19 presentation (e.g., date of positive test, need for hospitalization) from the EHR, curated the information, and entered the data into a secure REDCap database. For children with pre‐existing asthma, an additional data collection form was completed, which included baseline asthma severity (defined based on National Asthma Education and Prevention Program Guidelines [10], asthma medications, Asthma Control Test [11] (ACT; ages ≥ 12 years old) or Childhood‐Asthma Control Test [12] (C‐ACT; ages 4−11 years old) scores, spirometry results (percent‐predicted [%pred] using 2012 Global Lung Initiative equations [13], and whether the child had an asthma exacerbation during their acute COVID‐19 presentation. The Western Pennsylvania COVID‐19 Registry and this follow‐up study were approved by the University of Pittsburgh Institutional Review Board (protocols STUDY2011072 and STUDY22040031) with a waiver of informed consent, as there no research or study procedures (i.e., all data were extracted from the EHR).

Similar to our earlier study, we included children in the Western Pennsylvania COVID‐19 Registry who (1) were 6−21 years old, (2) had a diagnosis of asthma before SARS‐CoV‐2 infection, and (3) had asthma symptom control scores (ACT/C‐ACT) and/or spirometry both before COVID‐19 (“baseline”) and at least once after COVID‐19 (“follow‐up”). Subjects were excluded if they had significant comorbidities such as cystic fibrosis or bronchiectasis. For this analysis, we re‐reviewed the EHR for all children with asthma in the Western Pennsylvania COVID‐19 Registry who had baseline ACT/C‐ACT scores and/or spirometry and updated the REDCap database to include all post‐COVID‐19 results through August 2023.

### 2.2 Statistical Analysis

Our primary outcomes of interest were change in asthma symptom control, forced vital capacity (FVC %pred), forced expiratory volume in the first second (FEV_1_ %pred), and FEV_1_/FVC before and at latest follow‐up after COVID‐19. Because the ACT and C‐ACT have different ranges, we adopted a linear transformation for ACT score before combining it with C‐ACT score, as done previously [7, 14], so that they could be analyzed collectively: ACT′ = c*(ACT‐a)/(b‐a), where a denotes the ACT minimum score; b denotes the ACT maximum score; and c denotes the C‐ACT maximum score. Once ACT was transformed, it was combined with C‐ACT data, collectively referred to as “ACTS” (which includes “transformed ACT scores” and “C‐ACT scores”). We examined the change in asthma symptom control and lung function between baseline and final follow‐up visit using paired *t*‐tests. We also conducted subgroup analyses by COVID variant wave, which was defined (as done previously) based on the predominant local circulating SARS‐CoV‐2 variant during that period [15]. We conducted longitudinal mixed models of ACTS or FEV_1_ over time, adjusting for age, sex, race, and baseline severity, and with a linear spline, to evaluate trajectories within 500 days before versus 500 days after COVID‐19.

We then conducted a subgroup of analysis among children whose asthma symptom control or lung function had worsened at first post‐infection follow‐up. We defined worse asthma symptom control as a decrease in ACTS score of ≥ 3 points [16] and worse lung function as a decrease in FEV_1_ of ≥ 5 %pred. We compared characteristics of those whose symptoms or FEV_1_ subsequently returned to baseline (by their final follow‐up) vs those whose symptoms or FEV_1_ had not returned to baseline at final follow‐up. We also compared characteristics of those whose symptoms or FEV_1_ partially recovered (i.e., ACTS within 2 points of baseline and FEV_1_ within 3 %pred of baseline, respectively) at any subsequent follow‐up visit (i.e., not necessarily the *final* follow‐up visit). Finally, we compared characteristics of children without post‐COVID‐19 deficits in symptom control or lung function to those with post‐COVID‐19 deficits who did not recover. Bivariate analyses were conducted using *t* test, Wilcoxon Rank Sum test, Chi‐squared test, or Fisher Exact test. Analyses were conducted in SAS Version 9.4 (SAS Institute, Cary, North Carolina) or STATA v16.1 (StataCorp, College Station, TX).

## 3 Results

There were 3120 children in the Western Pennsylvania COVID‐19 Registry, of which 453 had asthma. Our final study cohort included 267 children with baseline and ≥ 1 post‐COVID‐19 ACTS score, and 196 children with baseline and ≥ 1 post‐COVID‐19 spirometry. SARS‐CoV‐2 infections occurred between 03/17/2020 and 01/24/2023 in our study population. Characteristics of the study populations are shown in Table 1.

**Table 1:** Characteristics of asthma symptom control study population and of spirometry study population.

	Asthma symptom control (ACTS^d^ score) *n* = 267	Spirometry *n* = 196
Age at time of COVID‐19 infection (years)	12.7 ± 3.8	12.0 ± 3.7
Period of infection‐ “variant wave”^a^		
7/1/2020 to 6/30/2021 (Pre‐Delta)	93 (35.6)	37 (19.4)
8/1/2021 to 12/14/2021 (Delta)	65 (24.9)	58 (30.4)
12/15/2021 to 8/30/2022 (Omicron)	103 (39.5)	96 (50.3)
Male sex	153 (57.3)	118 (60.2)
Race		
White	197 (75.5)	142 (73.6)
Black	58 (22.2)	50 (25.9)
Other	6 (2.3)	1 (0.5)
Body mass index percentile	79.7 [45.0−96.0]	81.2 [44.0−96.7]
Overweight or obese^b^	112 (43.2)	86 (45.5)
Asthma severity at pre‐infection baseline		
Intermittent	100 (37.5)	33 (16.8)
Mild persistent	97 (36.3)	87 (44.4)
Moderate persistent	55 (20.6)	51 (26.0)
Severe persistent	14 (5.2)	21 (10.7)
Asthma controller meds at pre‐infection baseline		
None	96 (36.0)	20 (10.2)
Montelukast	64 (24.0)	60 (30.6)
Inhaled corticosteroid	117 (43.8)	117 (59.7)
Combination (ICS‐LABA) inhaler	39 (14.6)	53 (27.0)
Asthma exacerbation with COVID‐19 infection^c^	60 (25.0)	57 (29.0)
Hospitalized with COVID‐19 infection	9 (3.4)	12 (6.2)
Total number of post‐infection follow‐ups		
1	131 (49.1)	73 (37.2)
2	102 (38.2)	59 (30.1)
3	25 (9.4)	29 (14.8)
4	5 (1.9)	14 (7.1)
5	3 (1.1)	9 (4.6)
6	1 (0.4)	6 (3.1)
7 or more	0 (0)	6 (3.1)
Time to last follow‐up (months)	11.4 [7.5−17.0] (range = 0.4−34.7)	11.4 [6.5−17.5] (range = 0.4−34.7)

*Note:* Results shown as average ± standard deviation, *N* (%), or median [Q1−Q3]. Range shown in () as indicated.

Abbreviations: ACT = Asthma Control Test, ACTS = transformed ACT score and C‐ACT score (collectively), C‐ACT = Childhood Asthma Control Test, ICS = inhaled corticosteroid, LABA = long‐acting beta‐agonist.

^a^ Variant wave, which was determined based on predominant circulating SARS‐CoV‐2 variant locally during that time period [15], was missing for 6/267 individuals with ACTS score and 5/196 individuals with spirometry, secondary to test date falling outside the windows used to define the variant waves;

^b^ Overweight or obese defined as body mass index *Z*‐score ≥ 85th percentile;

^c^ Data available for 240 individuals with ACT or C‐ACT data and 189 individuals with spirometry data;

^d^ Because the ACT and C‐ACT have different ranges, we adopted a linear transformation for ACT score before combining it with C‐ACT score, as follows: ACT′ = c × (ACT−a)/(b−a), where a denotes the ACT minimum score, b denotes the ACT maximum score, and c denotes the C‐ACT maximum score. Once ACT was transformed, it was combined with C‐ACT data, collectively referred to as “ACTS.”

For children with asthma symptom control results, mean age at time of infection was 12.7 years, 57% were male, 76% were White, and 43% were overweight or obese. Asthma severity ranged from mild intermittent (38%) to moderate/severe persistent (26%); 25% of participants had an asthma exacerbation during their acute SARS‐CoV‐2 infection, and 3% were hospitalized during their acute infection. The number of post‐infection follow‐ups ranged from 1 to 6; final follow‐up occurred a median of 11.4 months after COVID‐19 (range 0.4 to 34.7 months).

The population with spirometry results was similar. Mean age at time of SARS‐CoV‐2 infection was 12.0 years. Most (60%) were male, White (74%), 46% were overweight or obese, 17% had mild intermittent asthma, and 37% had moderate/severe persistent asthma; 29% of participants experienced an asthma exacerbation during their acute SARS‐CoV‐2 infection, and 6% were hospitalized during their acute infection. The number of post‐infection follow‐ups ranged from 1 to 13; final follow‐up occurred a median of 11.4 months after COVID‐19 infection (range 0.4−34.7).

**Figure 1:**
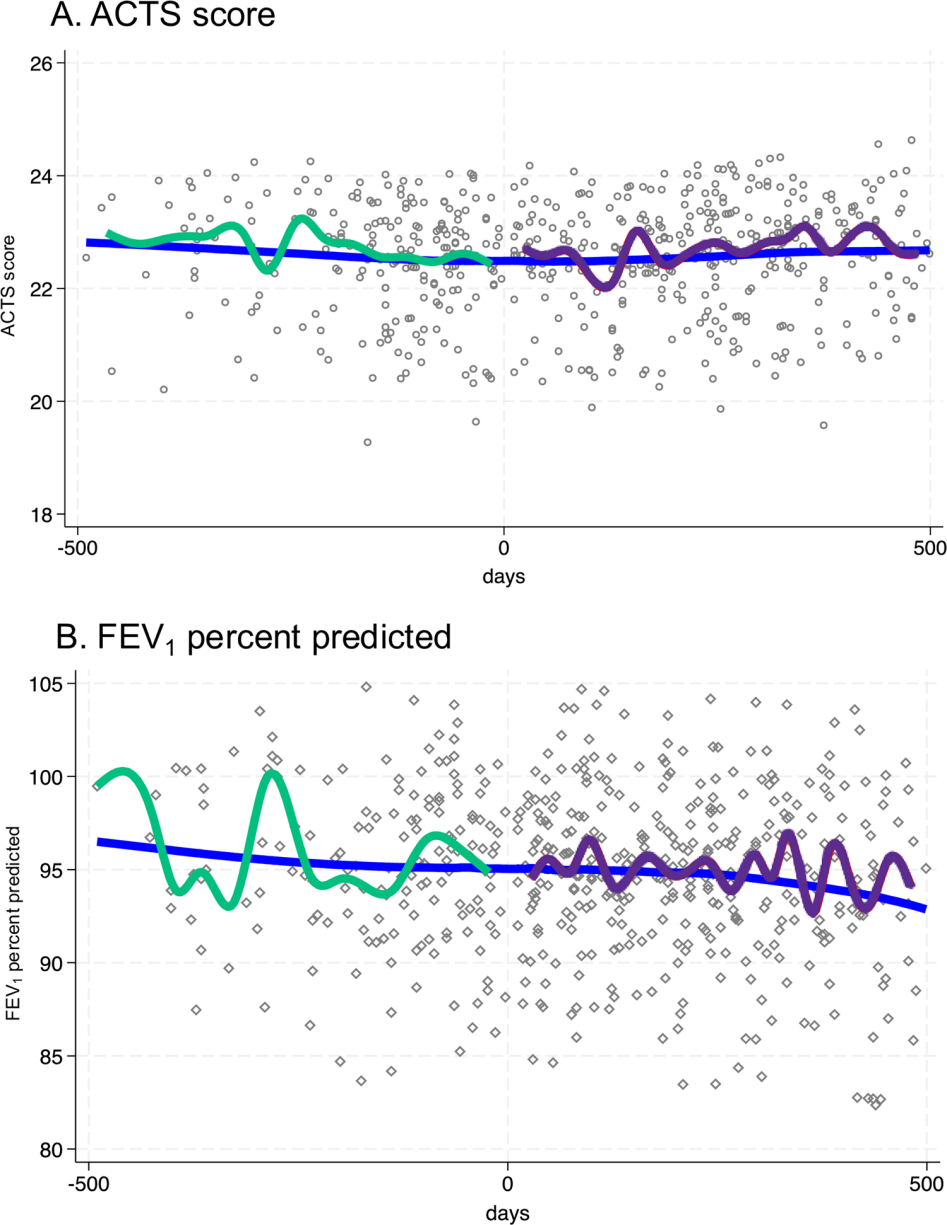
Adjusted mixed models with linear spline for ACTS score (Panel A) and FEV_1_ percent predicted (Panel B). Day 0 represents date of SARS‐CoV‐2 diagnosis. Panel A: When plotting all fitted values within ±500 days of their acute COVID‐19 infection, Lowess line and the median spline curves showed no significant changes in transformed ACT score before vs after COVID‐19 (*p* > 0.20). (Panel B): When plotting all fitted values within ±500 days of their acute COVID‐19 infection (see Figure), Lowess line and the median spline curves showed no significant changes in FEV_1_ percent predicted before vs after COVID‐19 (*p* > 0.20). [Color figure can be viewed at wileyonlinelibrary.com]

In paired analysis, there were no significant differences between baseline and final follow‐up ACTS score, FEV_1_ %pred, FVC %pred, or FEV_1_/FVC (Table 2), including by variant wave (Supporting Information S1: E‐Table 1). Average change in ACTS score between baseline and final follow‐up was 0.0 ± 4.7 points (*p* = 0.60), and average change in FEV_1_ was 0.0 ± 11.5 %pred (*p* = 0.99). The adjusted mixed models with linear spline showed no difference in slopes before vs after COVID‐19 for either ACTS or FEV_1_ (in both cases *p* > 0.20). When plotting all fitted values within ±500 days of their acute COVID‐19 infection (Figure 1), Lowess line and the median spline curves showed no significant changes in ACTS (Panel A) or FEV_1_ (Panel B) before vs after COVID‐19.

**Table 2:** Asthma symptom control and lung function before and after SARS‐CoV‐2 infection.

	Baseline	Final follow‐up^a^	Change^b^	*p* value
Asthma symptom control (*n* = 267)				
ACTS score^c^	23.0 [20.3−26.0]	24.0 [21.0−27.0]	0.0	0.60
Spirometry (*n* = 196)				
FEV_1_ %predicted	94.0 ± 18.1	94.0 ± 15.8	0.0 ± 11.5	0.99
FVC % predicted	101.2 ± 16.9	101.3 ± 16.7	0.1 ± 11.0	0.86
FEV1/FVC	82.1 ± 9.5	81.2 ± 8.5	−0.8 ± 7.1	0.13

*Note:* Results shown as average ± standard deviation or median [Q1–Q3]. *p* values are shown for paired *t*‐tests comparing baseline and final follow‐up ACTS score, FEV_1_ %pred, FVC %pred, or FEV1/FVC.

Abbreviations: ACT= Asthma Control Test, ACTS = transformed ACT score and C‐ACT score (collectively), C‐ACT = Childhood Asthma Control Test, FVC = forced vital capacity, FEV_1_ = forced expiratory volume over the first second.

^a^ Final follow‐up occurred a median of 11.4 months after SARS‐CoV‐2 infection;

^b^ “Change” was defined as the difference between final follow‐up and baseline measures for an individual;

^c^ Because the ACT and C‐ACT have different ranges, we adopted a linear transformation for ACT score before combining it with C‐ACT score, as follows: ACT′ = c × (ACT−a)/(b−a), where a denotes the ACT minimum score, b denotes the ACT maximum score, and c denotes the C‐ACT maximum score. Once ACT was transformed, it was combined with C‐ACT data, collectively referred to as “ACTS.”

Among children with ACTS data, 136/267 (50.9%) had ≥ 2 post‐COVID‐19 follow‐up ACTS scores and thus could be assessed for longitudinal post‐COVID‐19 asthma symptom control. Of the 50 children (18.7%) with worse ACTS score (i.e., ACTS score had decreased by least 3 points from baseline) at first follow‐up, 26 (52%) had ≥ 1 additional post‐infection ACTS score (Table 3). Of these 26 children, 10 (38%) fully recovered to baseline ACTS score at final follow‐up, while 16 (62%) did not. Compared to children who fully recovered to baseline ACTS score at final follow‐up, those who did not recover had higher baseline BMI percentile (93.9 vs. 49.0, *p* = 0.01), were more likely to be overweight or obese at baseline (90% vs. 11%, *p* = 0.04), and had higher baseline ACTS score (25 vs. 23, *p* = 0.04) and lower final follow‐up ACTS score (21.8 vs. 24.2, *p* = 0.01). While final ACTS score was lower for those who did not fully recover, the median magnitude of change in ACTS score between baseline and final follow‐up was only −3.0 [−4.7 to −1.7] points. Children with impaired recovery were also more likely to not be on a controller medication at baseline (37.5% vs. 0%), though this difference was not statistically significant (*p* = 0.05). There were no significant differences in age, race, sex, asthma severity, having an asthma exacerbation during COVID‐19 infection, or being hospitalized for COVID‐19 between those who did and those who did not have full recovery of ACTS score at final follow‐up.

**Table 3:** Characteristics of individuals who had worse asthma symptom control^a^ at initial post‐SARS‐CoV‐2 follow‐up, by recovery status at the time of their final follow‐up visit.

	ACTS score back to baseline by final follow‐up?		
	Yes (*n* = 10)	No (*n* = 16)	*p* value
Months between COVID‐19 infection and *first* follow‐up	4.6	3.0	0.45
	[2.1**–**7.6]	[1.0**–**6.8]	
Months between COVID‐19 infection and *final* follow‐up	20.3 [8.3**–**20.3]	14.8 [10.8**–**18.3]	0.75
Total number of post‐infection follow‐ups			1.00
2	2 (20.0)	13 (81.3)	
3	8 (80.0)	3 (18.8)	
4+	0	0	
Age at time of COVID‐19 infection (years)	11.9 [9.2**–**15.1]	10.6 [8.6**–**14.2]	0.58
Male sex	7 (70.0)	11 (68.8)	1.00
Race			1.00
Black	2 (22.2)	3 (21.4)	
White	7 (77.8)	11 (78.6)	
Body mass index percentile at baseline	49.0 [41.0–63.0]	93.9 [79.6–97.9]	0.01
Overweight or obese at baseline	1 (11.1)	9 (90.0)	0.04
Asthma severity at baseline			0.90
Intermittent	3 (30.0)	7 (43.7)	
Mild persistent	5 (50.0)	7 (43.7)	
Moderate persistent	1 (10.0)	1 (6.25)	
Severe persistent	1 (10.0)	1 (6.25)	
Baseline controller medication			
None	0 (0)	6 (37.5)	0.05
Inhaled corticosteroid	8 (80.0)	7 (43.8)	0.11
Combination (ICS‐LABA) inhaler	2 (20.0)	2 (12.5)	0.63
Leukotriene inhibitor	1 (10.0)	3 (18.8)	1.00
Asthma exacerbation with COVID‐19 infection^b^	3 (33.3)	7 (53.9)	0.41
Hospitalized with COVID‐19 infection	0 (0)	1 (6.3)	1.00
ACTS score at baseline	23.0 [20.0–24.3]	25.0 [23.7–26.5]	0.04
ACTS score at final follow‐up	24.2 [22.0–27.0]	21.8 [19.5–23.0]	0.01
Change in ACTS score between baseline and final follow‐up	1.2 [1.0–4.0]	−3.0 [−4.7 to −1.7]	< 0.001
Highest ACTS at any subsequent follow‐up visit	24.2 [22.0–27.0]	22.0 [20.8–23.0]	0.03
Change in ACTS between baseline and highest subsequent follow‐up	1.2 [1.0–4.0]	−3.0 [−4.1 to −1.7]	< 0.001

*Note:* Values in the table represent median [IQR] or *n* (%). *p* values are shown for the results of Wilcoxon Rank Sum test, Chi‐squared test, or Fisher′s Exact Test (based on variable type and distribution).

Abbreviations: ACT = Asthma Control Test, ACTS = transformed ACT score and C‐ACT score (collectively), C‐ACT = Childhood Asthma Control Test, ICS = inhaled corticosteroid, LABA = long‐acting beta‐agonist.

^a^ “Worse asthma symptom control” was defined as first post‐infection ACTS score being at least 3 points below baseline (pre‐infection) ACTS score;

^b^ Data available for 22/26 participants.

We also examined characteristics associated with “partial recovery,” defined as ACTS score recovering to within 2 points of baseline at any point after initial follow‐up (Supporting Information S1: E‐Table 2). 15/26 (58%) participants had partial recovery of ACTS score. As expected, ACTS score at final follow‐up and highest ACTS score at any subsequent follow‐up were higher among those who partially recovered (medians 24.0 vs. 21.6, *p* = 0.005 and 24.0 vs. 21.6, *p* = 0.01, respectively), but no other characteristics we examined were associated with partial recovery at any subsequent follow‐up visit.

Among children with baseline and follow‐up spirometry results, 123/196 (62.8%) had ≥ 2 post‐COVID‐19 follow‐up spirometry results and thus could have lung function assessed longitudinally after COVID‐19. Of the 55 children (28.1%) with worse FEV_1_ (i.e., FEV_1_ had decreased by > 5 %pred from baseline) at first follow‐up, 32 (58%) had ≥ 1 additional post‐infection follow‐up spirometry (Table 4). Of these 32 children, 11 (34%) recovered to baseline FEV_1_ at final follow‐up and 21 (66%) did not. There were no significant differences in age, sex, race, BMI percentile, asthma severity, having an asthma exacerbation during COVID‐19 infection, being hospitalized for COVID‐19, baseline FEV_1_, and final follow‐up FEV_1_ between those who did and those who did not have full FEV_1_ recovery. However, FEV_1_ increased between baseline and final follow‐up for those who fully recovered (mean change 4.6 ± 5.0 %pred) and decreased for those who did not (mean change −11.2 ± 8.2 %pred), *p* < 0.001.

**Table 4:** Characteristics of individuals who had worse lung function^a^ at initial post‐SARS‐CoV‐2 follow‐up, by recovery status at the time of their final follow‐up.

	FEV_1_ back to baseline by final follow‐up?		
	Yes (*n* = 11)	No (*n* = 21)	*p* value
Months between COVID‐19 infection and *first* follow‐up	2.6 [1.5−4.0]	5.4 [1.9−6.9]	0.13
Months between COVID‐19 infection and *final* follow‐up	16.1 [10.6−19.1]	17.9 13.1−22.0]	0.48
Total number of post‐infection follow‐ups			0.18
2	2 (18.2)	11 (52.4)	
3	5 (45.5)	2 (9.52)	
4+	4 (36.4)	8 (38.1)	
Age at time of COVID‐19 infection (years)	11.9 [9.9−14.8]	11.3 [8.7−14.0]	0.78
Male sex	7 (63.6)	13 (61.9)	1.00
Race			1.00
Black	2 (18.2)	3 (14.3)	
White	9 (81.8)	18 (85.7)	
Body mass index percentile at baseline	70.6 [38.9−92.0]	95.0 [56.0−98.0]	0.11
Overweight or obese at baseline	4 (40.0)	13 (61.9)	0.44
Asthma severity at baseline			0.67
Intermittent	0 (0)	1 (4.8)	
Mild persistent	7 (63.4)	9 (42.9)	
Moderate persistent	2 (18.2)	6 (28.6)	
Severe persistent	2 (18.2)	5 (23.8)	
Baseline controller medication			
None	0 (0)	1 (4.8)	1.00
Inhaled corticosteroid	6 (54.6)	10 (47.6)	1.00
Combination (ICS‐LABA) inhaler	3 (27.3)	9 (42.9)	0.47
Leukotriene inhibitor	5 (45.5)	6 (28.6)	0.44
Asthma exacerbation with COVID‐19 infection	3 (27.3)	5 (23.8)	0.83
Hospitalized with COVID‐19 infection	0 (0)	2 (9.5)	0.53
FEV_1_ at baseline (% predicted)	96.3 ± 14.0	103.3 ± 11.5	0.08
FEV_1_ at final follow‐up (% predicted)	100.9 ± 13.8	92.1 ± 14.5	0.16
Change in FEV_1_ between baseline and final follow‐up (% predicted)	4.6 ± 5.0	−11.2 ± 8.2	< 0.001
Highest FEV_1_ at any subsequent follow‐up visit (% predicted)	101.4 ± 14.8	98.4 ± 10.1	0.62
Change in FEV_1_ between baseline and highest subsequent follow‐up (% predicted)	5.0 ± 5.0	−4.9 ± 6.1	< 0.001

*Note:* Values in the table represent mean ± standard deviation, median [IQR], or *n* (%). *p* values are shown for the results of *t*‐test, Wilcoxon Rank Sum test, Chi‐squared test, or Fisher′s Exact Test (based on variable type and distribution).

Abbreviations: ICS = inhaled corticosteroid, LABA = long‐acting beta‐agonist.

^a^ “Worse lung function” was defined as first post‐infection FEV_1_ being at least 5% predicted lower than baseline (pre‐infection) FEV_1_.

We also examined characteristics associated with “partial recovery,” defined as FEV_1_ %pred recovering to within 3 %pred of baseline at any point after initial follow‐up (Supporting Information S1: E‐Table 3). 18/32 (56%) participants had partial recovery of FEV_1_. Participants with partial recovery were more likely to have had > 2 post‐infection follow‐ups, and the change in FEV_1_ between baseline and highest subsequent (not initial) follow‐up was higher for those with partial recovery compared to those without partial recovery (3.7 ± 5.5 vs. −8.2 ± 3.6 %pred, *p* < 0.001). There were no other differences between those who did and those who did not have partial recovery in FEV_1_ at any subsequent follow‐up visit; highest FEV_1_ at any subsequent follow‐up visit was similar between those with and those without partial recovery (101.2 ± 13.8 vs. 97.1 ± 8.5 %pred, *p* = 0.39).

Children without post‐COVID‐19 decreases in ACTS or FEV_1_ were compared to children who had post‐COVID‐19 worsening without subsequent recovery (Table 5). Compared to those who did not have worse ACTS score post‐COVID‐19 (*n* = 217), those who had deficits without recovery (*n* = 16) were more likely to have had an asthma exacerbation during COVID‐19 (54% vs 22%, *p* = 0.02), had higher baseline ACTS score (median 25.0 vs. 23.0, *p* = 0.04), and had lower ACTS score at final follow‐up (median 21.8 vs. 25.0, *p* < 0.001). There were no differences between children without post‐COVID‐19 decreases in FEV_1_ and those who had FEV_1_ decreases without recovery.

**Table 5:** Comparison of characteristics of children without post‐COVID‐19 deficits in symptom control or lung function to those with post‐COVID‐19 deficits who never recovered.

	ACTS			FEV_1_		
	No post‐COVID‐19 deficits *n* = 217	Deficits that did not recover *n* = 16	*p* value	No post‐COVID‐19 deficits *n* = 141	Deficits that did not recover = 21	*p* value
Age at time of COVID‐19 infection (years)	13.0 [10.1−16.1]	10.6 [8.6−14.2]	0.05	12.2 [9.1−15.4]	11.3 [8.7−14.0]	0.48
Male sex	125 (57.6)	11 (68.8)	0.38	81 (57.5)	13 (61.9)	0.70
Race			0.90			0.33
Black	46 (21.5)	3 (21.4)		40 (29.0)	3 (14.3)	
White	165 (77.1)	11 (78.6)		97 (70.3)	18 (85.7)	
Body mass index percentile at baseline	79.4 [44.0−96.0]	93.9 [79.6−97.9]	0.11	81.2 [44.5−96.7]	95.0 [56.0−98.0]	0.28
Overweight or obese at baseline	93 (44.3)	9 (56.3)	0.35	61 (44.5)	13 (61.9)	0.14
Asthma severity at baseline			0.51			0.21
Intermittent	80 (36.9)	7 (43.8)		29 (20.6)	1 (4.8)	
Mild persistent	79 (36.4)	7 (43.8)		55 (39.0)	9 (42.9)	
Moderate persistent	46 (21.2)	1 (6.3)		39 (27.7)	6 (28.6)	
Severe persistent	11 (5.1)	1 (6.3)		14 (9.9)	5 (23.8)	
Baseline controller medication						
None	81 (37.3)	6 (37.5)	0.99	16 (11.4)	1 (4.8)	0.36
Inhaled corticosteroid	93 (42.9)	7 (43.8)	0.94	10 (47.6)	83 (58.9)	0.33
Combination (ICS‐LABA) inhaler	31 (14.3)	2 (12.5)	0.84	39 (27.7)	9 (42.9)	0.15
Leukotriene inhibitor	55 (25.4)	3 (18.8)	0.77	41 (29.1)	6 (28.6)	0.96
Hospitalized with COVID‐19 infection	7 (3.2)	1 (6.3)	0.52	9 (6.5)	2 (9.5)	0.61
Asthma exacerbation with COVID‐19 infection	43 (21.9)	7 (53.9)	0.02	43 (30.5)	98 (69.5)	0.53
ACTS at baseline	23.0 [20.0−26.0]	25.0 [23.7−26.5]	0.04			
ACTS at final follow‐up	25.0 [21.6−27.0]	21.8 [19.5−23.0]	< 0.001			
FEV_1_ at baseline (% predicted)				90.8 ± 18.5	103.3 ± 11.5	0.08
FEV_1_ at final follow‐up (% predicted)				93.7 ± 16.1	92.1 ± 14.5	0.57

*Note:* Values in the table represent mean ± standard deviation, median [IQR], or n (%). *p* values are shown for the results of *t*‐test, Wilcoxon Rank Sum test, Chi‐squared test, or Fisher's Exact Test (based on variable type and distribution).

Abbreviations: ACT= Asthma Control Test, ACTS = transformed ACT score and C‐ACT score (collectively), C‐ACT = Childhood Asthma Control Test, ICS = inhaled corticosteroid, LABA = long‐acting beta‐agonist.

## 4 Discussion

In this extended prospective study, we found no significant differences in symptom control or lung function up to 34 months (median 11 months) after SARS‐CoV‐2 infection in youth with asthma. This expands upon our prior study in which we found no significant differences in asthma symptom control or FEV_1_, FVC, or FEV_1_/FVC up to 18 months (median ~5 months) after COVID‐19 [7]. Similarly, a Turkish study of children with asthma found no significant differences in FEV_1_, FVC, or FEV_1_/FVC before versus up to 24 months (median ~16 months) after COVID‐19; however, that study found statistically, but not clinically, significant differences in pre‐ versus post‐COVID‐19 ACT (median 23 vs. 22.5, *p* = 0.001) and C‐ACT (median 25 vs. 25, *p* = 0.04) scores [17]. Notably, our study population with asthma symptom control data overall had milder asthma than our population with spirometry; this is likely attributable to the fact that while ACT and C‐ACT are routinely conducted in primary care settings in our area, spirometry is typically only obtained in specialty clinics (Pulmonology or Allergy).

Other studies have also reported that children with asthma do not have spirometry abnormalities after COVID‐19. A recent meta‐analysis including 8 studies found no abnormalities in pulmonary function tests in youth (with and without asthma) a median of ~1.5 to 10 months after COVID‐19 [18]. Though half of the studies in the meta‐analysis excluded children with asthma, meta‐regression found that having a diagnosis of asthma did not have a significant effect on the pooled mean of FEV_1_ and FVC [18]. Similarly, a study of 433 Italian children found that only 3.2% of had FEV_1_ < 80 %predicted an average of 5.7 months after COVID‐19; unsurprisingly, low FEV_1_ was significantly correlated with a history of asthma before COVID‐19 infection and thus may have been due to lower baseline FEV_1_ [19]. A significant limitation of these studies is that they did not include pre‐SARS‐CoV‐2 lung function as a baseline comparison, and they only included a single post‐infection pulmonary function test for each study participant. In contrast, our present study included pre‐infection baseline lung function testing results, and more than half of participants had > 1 post‐infection follow‐up lung function test. Among the 28% of children in our study who had worse lung function at initial follow‐up, 34% fully recovered at final follow‐up and 56% had partial recovery at any subsequent follow‐up, and reassuringly, there were no significant differences in highest follow‐up FEV_1_ based on recovery status.

Additionally, the maximum follow‐up timeframe in these studies was 18 months after infection [18, 19] and thus they were unable to provide information about long‐term effects of SARS‐CoV‐2 on pulmonary function tests in children. A small study of 34 children in Turkey found that only 12% had FEV_1_ < 80% predicted a median of 15 months (maximum 29 months) after hospitalization for COVID‐19 [20], and that having FEV_1_ < 80% predicted was significantly associated with having severe rather than moderate COVID‐19 disease (33% vs 4%, *p* = 0.048) [20]; however, that study excluded children with pre‐existing asthma. In our present study, we followed children with asthma for up to 34 months post‐infection. To our knowledge, this is the longest follow‐up period for a study of pulmonary function in children with asthma after SARS‐CoV‐2 infection.

While our present study is the first to examine pulmonary function test results in youth up to 34 months after COVID‐19, a recent study measured pulmonary function in adults hospitalized for COVID‐19 at 6, 12, 24, and 36 months after SARS‐CoV‐2 infection [21]. They found no significant difference in the proportion of individuals with FEV_1_ < 80% at each follow‐up time point, including when they stratified by COVID‐19 disease severity scale [21]. Interestingly, that study found a large proportion of participants (49%) had diffusion impairment, defined as DLCO < 80% predicted, at 6 months post‐infection, but this proportion gradually improved at subsequent follow‐ups (43% at 12 months, 40% at 24 months, 38% at 36 months; *p* = 0.001 comparing 6 months to 36 months) [21].

There is conflicting evidence regarding the impact of COVID‐19 on asthma symptom control in children with asthma. A small French study of 51 children with asthma who contracted COVID‐19 during a September 2020 outbreak found that asthma remained controlled in all children after infection [22], and our prior study found no significant differences in asthma symptom control before versus after COVID‐19 in youth with asthma [7]. However, a large EHR‐based study of > 61,000 youth with asthma in the United States found higher rates of ED visits, hospitalizations, oral corticosteroid fills, and short‐acting beta‐agonist fills among the 7746 children who tested positive for SARS‐CoV‐2 in the 6 months following SARS‐CoV‐2 testing, suggesting that SARS‐CoV‐2 infection is associated with increased asthma exacerbations in the months following acute infection [23]. Similarly, a study of Taiwanese youth with asthma found that children who tested positive for SARS‐CoV‐2 had lower C‐ACT scores 1 month after infection than age‐matched children with asthma without SARS‐CoV‐2 (median 25 vs. 27, *p* < 0.001) [24]; however, this study did not include pre‐SARS‐CoV‐2 C‐ACT scores for comparison, and these differences, while statistically significant, may not be clinically significant. As noted above, a study of Turkish youth with asthma found statistically significant differences in pre‐ versus post‐SARS‐CoV‐2 infection ACT (median 23 vs. 22.5, *p* = 0.001) and C‐ACT (median 25 vs. 25, *p* = 0.04) scores [17], but it is unclear if these differences are clinically meaningful, and they only obtained a single follow‐up measurement for each study participant.

In contrast to these prior studies of asthma control after pediatric COVD‐19, > 50% of participants in our present study had > 1 follow‐up assessments of asthma symptom control. While our study found that overall, there was no significant decrease in symptom control after COVID‐19, a subset of individuals (19%) had worse control at initial follow‐up. Of these, 38% had full recovery at final follow‐up and over half (58%) had partial recovery at any subsequent follow‐up, and fortunately, the decrements in final ACTS score for those who did not recover were fairly small (median −3.0 points). We also found that having an asthma exacerbation during COVID‐19 was associated with impaired ACTS recovery compared to those who did not have ACTS decline after COVID‐19, consistent with our prior study in which we found that children whose asthma control worsened after COVID‐19 were more likely to have presented with an asthma exacerbation during COVID‐19 than those without worse control [7]. The aforementioned Taiwanese study found that poor asthma control after SARS‐CoV‐2 infection was a risk factor anxiety and depression among children with asthma [24], highlighting the importance of further studying this subgroup with worse asthma symptom control after COVID‐19. This population with impaired recovery should also be studied in the context of pediatric long COVID, an area of ongoing research [25, 26]. It is possible that observed impairments in asthma symptom control may represent long COVID rather than worse asthma control.

BMI was the only characteristic we identified that significantly differed by asthma symptom control recovery status. Interestingly, obesity was associated with having a > 25% decrease in FEF25‐75 after SARS‐CoV‐2 infection in a study of Turkish youth with asthma, but that study did not assess if obesity was associated with lower post‐infection asthma symptom control [17]. Genes associated with ACE2, a known receptor for SARS‐CoV‐2 [27], have been shown to be expressed more highly in individuals with asthma and individuals with obesity [28]. CD147 is a potential novel route for SARS‐CoV‐2 entry [29], and CD147 gene expression has been positively correlated with BMI [28]. ACE2 and CD147 could thus be contributing to differential SARS‐CoV‐2 outcomes in youth with asthma and obesity, though this hypothesis requires further study‐ other pathophysiologic mechanisms may be driving our observed differences.

Our study had several strengths. To our knowledge, this is the first study assessing lung function and asthma symptom control in children with asthma at multiple time points after acute SARS‐CoV‐2 infection, compared to pre‐infection baseline. This provided the opportunity to follow the subgroup of children with worse asthma symptom control or lung function at initial follow‐up and to assess for characteristics associated with lack of eventual improvement. Additionally, our study has the longest follow‐up assessment period (up to 34 months) for children with asthma to date. We also acknowledge several important limitations. The number of participants with reduced lung function or asthma symptom control at first follow‐up was small, and the number with more subsequent follow‐ups was even smaller, providing limited power to detect characteristics associated with lack of eventual recovery. Future larger studies will be needed to better assess this important subgroup who does not recover to baseline lung function or symptom control, particularly given our observation that BMI and obesity were associated with non‐recovery of symptom control. Additionally, while we found no differences in follow‐up symptom control or lung function by SARS‐CoV‐2 variant wave, because of our small sample size of youth with reduced lung function or asthma symptom control at first follow‐up visit we were unable to examine recovery status by variant. This is an important consideration, given reports that the Omicron variant is associated with greater odds of asthma exacerbation and hospitalization among children with asthma than prior SARS‐CoV‐2 variants [15, 30]. Our study was an observational prospective chart review study and thus was subject to confounding and sampling bias, and we lacked data on some covariates of interest such as COVID‐19 vaccination status. Finally, our study was conducted at a single center, so our findings may not be generalizable to other populations of children with asthma, and we excluded children < 6 years old, so our findings may not be generalizable to toddlers or preschoolers with asthma.

In sum, in this long‐term follow‐up study of children with asthma and COVID‐19, we found no significant differences in asthma symptom control or lung function up to 34 months after infection compared to pre‐infection baseline. Of the subgroup of children with lower lung function or symptom control at first post‐COVID‐19 follow‐up, over half had at least partial recovery at subsequent follow‐up. Final follow‐up FEV_1_ did not differ by recovery status. While final follow‐up ACTS score was lower than pre‐infection baseline for those without recovery, the median score was still > 19, indicating adequate asthma control. BMI and obesity were associated with not having full recovery of asthma symptom control at final follow‐up.

## Author Contributions

**Kristina Gaietto:** conceptualization, data curation, formal analysis, methodology, investigation, supervision, writing − original draft, writing − review and editing. **Nicholas Bergum:** writing − review and editing, data curation, conceptualization, formal analysis. **Daniel J. Weiner:** data curation, writing – review and editing. **Erick Forno:** conceptualization, methodology, software, supervision, formal analysis, resources, project administration, visualization, investigation, writing – review and editing, data curation.

## Ethics Statement

This is approved by the Institutional Review Board at the University of Pittsburgh (protocol STUDY20110072).

## Conflicts of Interest

The authors declare no conflicts of interest.

## Supporting information

R1 OS COVID long follow up 073125.

## Data Availability

The data that support the findings of this study are available on request from the corresponding author. The data are not publicly available due to privacy or ethical restrictions.
