# Supplementary material for: Observational Study: Lung Function and Symptom Control in Youth With Asthma up to 34 Months After COVID‐19
Source: Pediatr Pulmonol. 2025 Sep 9;60(9):e71288. doi: 10.1002/ppul.71288 (PMC12418911; doi:10.1002/ppul.71288)
Supplement: Supplementary file 1 — R1 OS COVID long follow up 073125. [file PPUL-60-0-s001.docx]

**Online Supplementary Data**

**Observational Study: Lung Function and Symptom Control in Youth with Asthma Up to 34 Months After COVID-19**

Kristina Gaietto, MD MPH^†1^, Nicholas Bergum, MD^†2^, Daniel J. Weiner, MD^1^, Erick Forno, MD MPH^3^

*^1^Department of Pediatrics, Division of Pulmonology, University of Pittsburgh School of Medicine, Pittsburgh, PA; ^2^University of Pittsburgh School of Medicine, Pittsburgh, PA; ^3^Department of Pediatrics, Division of Pulmonology, Indiana University of School of Medicine, Indianapolis, IN*

^†^Denotes shared first authors

**E-table 1.** Change in asthma symptom control and lung function after SARS-CoV-2 infection by variant wave period

|  | **Pre-Delta wave**  (7/1/2020 to 6/30/2021) | **Delta wave**  (8/1/2021 to 12/14/2021) | **Omicron wave**  (12/15/2021 to 8/30/2022) | **ANOVA**  **p-value** |
| --- | --- | --- | --- | --- |
| **Asthma symptom control (n=267)** | n = 93 | n = 65 | n = 103 |  |
| Change^1^ in ACTS2 score | -0.12 ± 4.41 | 0.59 ± 3.93 | 0.20 ± 5.48 | 0.65 |
| Paired t test result for change in ACTS score | p=0.79 | p=0.23 | p=0.71 |  |
| **Spirometry (n=196)** | n = 37 | n = 58 | n = 96 |  |
| Change^1^ in FEV_1_ %predicted | -1.76 ± 10.65 | -0.05 ± 12.16 | 0.47 ± 11.41 | 0.61 |
| Paired t test result for change in FEV_1_ %predicted | p=0.32 | p=0.98 | p=0.69 |  |
| Change^1^ in FVC % predicted | -1.43 ± 8.27 | -0.13 ± 12.96 | 0.75 ± 10.59 | 0.58 |
| Paired t test result for change in FVC % predicted | p=0.30 | p=0.94 | p=0.49 |  |
| Change^1^ in FEV_1_/FVC | -0.99 ± 7.01 | -0.14 ± 6.74 | -1.23 ± 7.50 | 0.65 |
| Paired t test result for change in FEV_1_/FVC | p=0.40 | p=0.88 | p=0.11 |  |
| *Results shown as average ± standard deviation of change in outcome measure (ACTS or spirometry) between final follow-up and baseline. P-values are for paired t-tests comparing baseline and final follow-up ACTS score, FEV1 %pred, FVC %pred, or FEV1/FVC and for the ANOVA comparing the change between baseline and follow-up ACTS score, FEV1 %pred, FVC %pred, or FEV1/FVC.*  *^1^“Change” was defined as the difference between final follow up and baseline measures for an individual.*  *^2^Because the ACT and C-ACT have different ranges, we adopted a linear transformation for ACT score before combining it with C-ACT score, as follows: ACT' = c × (ACT − a)/(b − a), where a denotes the ACT minimum score, b denotes the ACT maximum score, and c denotes the C-ACT maximum score. Once ACT was transformed, it was combined with C-ACT data, collectively referred to as “ACTS”.*  *ACTS = transformed ACT score and C-ACT score (collectively), ACT= Asthma Control Test, C-ACT = Childhood Asthma Control Test, FVC = forced vital capacity, FEV_1_ = forced expiratory volume over the first second.* | | | | |

**E-table 2.** Characteristics of individuals who had worse asthma symptom control^1^ at initial post-SARS-CoV-2 follow-up, by at least partial recovery status at any subsequent follow-up visit

|  | ACTS score within 2 points of baseline at any subsequent follow up visit? | | |
| --- | --- | --- | --- |
|  | Yes  (n=15) | No  (n=11) | p-value |
| Months between COVID-19 infection and *first* follow-up | **4.5**  **[2.3-7.6]** | **1.9**  **[0.7-6.1]** | **0.048** |
| Months between COVID-19 infection and *final* follow-up | 16.9  [7.6-20.3] | 14.4  [13.2-19.4] | 0.86 |
| Total number of post-infection follow-ups |  |  | 0.62 |
| 2 | 13 (86.7) | 8 (72.7) |  |
| 3 | 2 (13.3) | 3 (27.3) |  |
| 4+ | 0 | 0 |  |
| Age at time of COVID-19 infection (years) | 11.7  [9.2-15.1] | 11.0  [7.8-14.2] | 0.64 |
| Male sex | 11 (73.3) | 7 (63.6) | 0.68 |
| Race |  |  | 1.00 |
| Black | 3 (21.4) | 2 (22.2) |  |
| White | 11 (78.6) | 7 (77.8) |  |
| Body mass index percentile at baseline | 58.1  [41.0-95.0] | 92.1  [79.2-98.6] | 0.06 |
| Overweight or obese at baseline | 4 (28.6) | 6 (54.6) | 0.24 |
| Asthma severity at baseline |  |  | 0.56 |
| Intermittent | 4 (26.7) | 6 (54.6) |  |
| Mild persistent | 8 (53.3) | 4 (36.4) |  |
| Moderate persistent | 2 (13.3) | 0 (0) |  |
| Severe persistent | 1 (6.7) | 1 (9.1) |  |
| Baseline controller medication |  |  |  |
| None | **1 (6.7)** | **5 (45.5)** | **0.05** |
| Inhaled corticosteroid | 11 (73.3) | 4 (36.4) | 0.11 |
| Combination (ICS-LABA) inhaler | 2 (13.3) | 2 (18.2) | 1.00 |
| Leukotriene inhibitor | 2 (13.3) | 2 (18.2) | 1.00 |
| Asthma exacerbation with COVID-19 infection^2^ | 5 (38.5) | 5 (55.6) | 0.67 |
| Hospitalized with COVID-19 infection | 1 (6.7) | 0 (0) | 1.00 |
| ACTS score at baseline | 23.0  [20.0-25.0] | 25.7  [23.0-27.0] | 0.06 |
| ACTS score at final follow-up | **24.0**  **[22.0-25.7]** | **21.6**  **[11.0-22.0]** | **0.005** |
| Change in ACTS score between baseline and final follow-up | **1.0**  **[-1.0 – 2.7]** | **-4.1**  **[-6.8 - -3.0]** | **<0.001** |
| Highest ACTS at any subsequent follow-up visit | **24.0**  **[22.0 – 25.7]** | **21.6**  **[20.0 – 22.0]** | **0.01** |
| Change in ACTS between baseline and highest subsequent follow-up | **1.0**  **[-1.0 – 2.70]** | **-4.0**  **[-5.4 - -3.0]** | **<0.001** |
| *Values in the table represent median [IQR] or n (%). p-values are shown for the results of Wilcoxon Rank Sum test, Chi-squared test, or Fisher’s Exact Test (based on variable type and distribution).*  *^1^“Worse asthma symptom control” was defined as first post-infection ACTS score being at least 3 points below baseline (pre-infection) ACTS score.*  *^2^Data available for 22/26 participants.*  *ACTS = transformed ACT score and C-ACT score (collectively), ACT = Asthma Control Test, C-ACT = Childhood Asthma Control Test, ICS = inhaled corticosteroid, LABA = long-acting beta-agonist* | | | |

**E-table 3.** Characteristics of individuals who had worse lung function^1^ at initial post-SARS-CoV-2 follow-up, by at least partial recovery status at any subsequent follow-up visit

|  | FEV_1_ within 3 %predicted of baseline at any subsequent follow up visit? | | |
| --- | --- | --- | --- |
|  | Yes  (n=18) | No  (n=14) | p-value |
| Months between COVID-19 infection and *first* follow-up | 2.6  [1.5-4.1] | 5.9  [3.0-10.0] | 0.08 |
| Months between COVID-19 infection and *final* follow-up | 17.6  [13.6-19.1] | 17.6  [8.0-22.2] | 1.00 |
| Total number of post-infection follow-ups |  |  | **<0.001** |
| 2 | **3 (16.7)** | **10 (71.4)** |  |
| 3 | **7 (38.9)** | **0 (0.0)** |  |
| 4+ | **8 (44.4)** | **4 (28.6)** |  |
| Age at time of COVID-19 infection (years) | 11.9  [9.2-13.6] | 10.8  [8.7-15.1] | 0.78 |
| Male sex | 9 (50.0) | 11 (78.8) | 0.15 |
| Race |  |  | 1.00 |
| Black | 3 (16.7) | 2 (14.3) |  |
| White | 14 (83.3) | 12 (85.7) |  |
| Body mass index percentile at baseline | 66.0  [40.6-94.0] | 95.9  [67.0-98.0] | 0.12 |
| Overweight or obese at baseline | 7 (41.2) | 10 (71.4) | 0.15 |
| Asthma severity at baseline |  |  | 0.53 |
| Intermittent | 1 (5.6) | 0 (0) |  |
| Mild persistent | 9 (50.0) | 7 (50.0) |  |
| Moderate persistent | 3 (16.7) | 5 (35.7) |  |
| Severe persistent | 5 (27.8) | 2 (14.3) |  |
| Baseline controller medication |  |  |  |
| None | 0 (0) | 1 (7.1) | 0.44 |
| Inhaled corticosteroid | 9 (50.0) | 7 (50.0) | 1.00 |
| Combination (ICS-LABA) inhaler | 7 (38.9) | 5 (35.7) | 1.00 |
| Leukotriene inhibitor | 6 (33.3) | 5 (35.7) | 1.00 |
| Asthma exacerbation with COVID-19 infection | 6 (33.3) | 12 (66.7) | 0.22 |
| Hospitalized with COVID-19 infection | 1 (5.6) | 1 (7.1) | 1.00 |
| FEV_1_ at baseline (% predicted) | 97.4 ± 14.1 | 105.3 ± 9.2 | 0.06 |
| FEV_1_ at final follow-up (% predicted) | 96.7 ± 18.2 | 93.1 ± 8.6 | 0.26 |
| Change in FEV_1_ between baseline and final follow-up (% predicted) | -0.7 ± 9.7 | -12.2 ± 7.5 | **0.001** |
| Highest FEV_1_ at any subsequent follow-up visit (% predicted) | 101.2 ± 13.8 | 97.1 ± 8.5 | 0.39 |
| Change in FEV_1_ between baseline and highest subsequent follow-up (% predicted) | 3.7 ± 5.5 | -8.2 ± 3.6 | **<0.001** |
| *Values in the table represent mean ± standard deviation, median [IQR], or n (%). p-values are shown for the results of t-test, Wilcoxon Rank Sum test, Chi-squared test, or Fisher’s Exact Test (based on variable type and distribution).*  *^1^“Worse lung function” was defined as first post-infection FEV_1_ being at least 5 % predicted lower than baseline (pre-infection) FEV_1_.*  *ICS = inhaled corticosteroid, LABA = long-acting beta-agonist* | | | |
